# Worsening of asthma control after COVID-19

**DOI:** 10.3389/fmed.2022.882665

**Published:** 2022-09-14

**Authors:** Rosana Câmara Agondi, Natália Menechino, Ana Karolina Barreto Berselli Marinho, Jorge Kalil, Pedro Giavina-Bianchi

**Affiliations:** ^1^Clinical Immunology and Allergy Division, Department of Medical Clinic, School of Medicine, University of São Pãulo, São Paulo, Brazil; ^2^Laboratory of Immunology (LIM19), School of Medicine, Heart Institute (InCor), University of São Paulo, São Paulo, Brazil

**Keywords:** asthma, COVID-19, asthma control, post-COVID-19 condition, atopy, comorbidities, T2 asthma

## Abstract

**Background:**

SARS-CoV-2 enters lung cells *via* angiotensin-converting enzyme 2 (ACE2) receptor. Several studies suggest that interleukin-13, an important cytokine involved in T2 inflammation, reduces ACE2 expression, and therefore, asthma would not be a significant risk factor for the development of severe COVID-19. However, several asthma-related risk factors should be valued during the concurrent occurrence of asthma and COVID-19. The purpose of this study was to compare the evolution of asthma in patients who had COVID-19 with those who did not have the disease.

**Methods:**

This was an observational and retrospective study involving asthmatic patients followed up at a tertiary center. Patients were assessed for severity of asthma, atopy, comorbidities, and COVID-19. Worsening of asthma was considered when, during the period of Sept 2020 to Oct 2021, patients referred an increasing of asthma symptoms and a need to increment their maintenance therapy.

**Results:**

This study included 208 asthmatic patients, the mean age was 52.75 years, 79.81% were atopic asthmatics, and 59 (28.37%) had laboratory-confirmed coronavirus disease. Of all patients infected with the SARS-CoV-2, eleven (18.64%) needed hospitalization and required oxygen supply with an O2 mask. Comparing the worsening of asthma between patients who had COVID-19 and those who had not the disease, there was a statistically significant difference, 33.90 vs. 11.41%, respectively (*p* < 0.001). There was no statistical significance regarding asthma comorbidities.

**Conclusion:**

This study assessed a group of asthmatic patients that had COVID-19, and that although the respiratory symptoms related to COVID-19 were mild to moderate, a subgroup of these asthmatic patients evolved with a chronic worsening of their asthma requiring an increment in asthma medication to control the disease.

## Introduction

Asthma is one of the most prevalent chronic respiratory diseases in the world, asthma exacerbation may be life-threatening and carry a significant burden to patients and the community. Asthma exacerbations are often triggered by multiple factors, including virus infection. Several studies showed that patients with asthma have an increased susceptibility to viruses, bacteria or fungi infections (1, 2). Viral respiratory infections, primarily from rhinoviruses, are the dominant exacerbating cause for most asthma patients, adults and school-aged children. Allergic sensitization and allergen exposure contribute directly and enhance susceptibility for respiratory viral infections. One key interlinking areas between asthma exacerbation and respiratory viruses relates to inappropriate T2 inflammatory mediators. The epithelial-derived cytokine interleukin (IL)-33 synthesized in response to bronchial epithelial infection appears to be a particularly important mediator driven T2 response to rhinovirus and other viruses (3).

SARS-CoV-2 (Severe Acute Respiratory Syndrome Coronavirus 2) uses angiotensin-converting enzyme 2 (ACE2) as its cellular receptor. ACE2 expression was significantly and inversely associated with type 2 biomarkers, i.e., lower levels were observed in patients with allergic sensitization and asthma. Several studies suggested that IL-13, an important cytokine involved in T2 inflammation, could play a role in this mechanism, as it reduces ACE2 expression *ex vivo* in airway epithelial cells (4, 5).

Although there is variable asthma prevalence among published studies of COVID-19, the prevalence appears to be similar to the general population and is certainly much lower than what would be expected during seasonal influenza (6). Broadhurst et al. (6) suggested that asthma does not appear to be a significant risk factor for developing severe COVID-19 requiring hospitalization or intubation.

According to World Health Organization (WHO), COVID-19 may cause symptoms that will last weeks or months after the infection has gone, and this condition is called “long-COVID-19.” Regarding COVID-19, ~10–15% of the patients will progress to severe disease and 5% become critically ill, although, the majority recovers from COVID-19 after 2 to 6 weeks. Long-COVID-19 occurs in individuals with a history of probable or confirmed SARS-CoV-2 infection, usually 3 months from the onset of symptomatic COVID-19, that last for at least 2 months and cannot be explained by an alternative diagnosis (7). Common symptoms include chest pain, breathlessness, muscle weakness, anxiety, depression, palpitations, abdominal pain, and, fatigue; which is thought to affect 10% of those diagnosed with COVID-19 (7–9).

In general, there is neither consistent evidence that patients with asthma are at greater risk of becoming infected by SARS-CoV-2 nor that they are at greater risk of having poor outcomes of COVID-19. There is evidence of reduced hospitalizations because of asthma during the pandemic, which is likely an effect of reduced frequency of acute respiratory infections because of behavior aiming at COVID-19 prevention. Treatment of patients with asthma and rhinitis must be optimized to minimize the requirement for oral corticosteroids and need for emergency care. By other hand, there are reports indicating that patients with asthma may be at lower risk of hospitalizations during the COVID-19 pandemic (10).

One systematic review and meta-analysis showed that the presence of co-morbidities such as diabetes, hypertension, cardiovascular disease, chronic obstructive pulmonary disease in patients with COVID-19 were associated with an increased risk of developing severe symptoms and mortality (11).

The major objective of this study was to compare the evolution of asthma in patients who had COVID-19 with those who did not have the disease.

## Materials and methods

This was an observational and retrospective study involving asthmatic patients who have been followed up at the Asthma Outpatient Clinic of the Clinical Immunology and Allergy Division, University of São Paulo, Brazil. This study was approved by the Ethics Committee of University of São Paulo, Medical School (CAPPesq), under the number 36479220.0.0000.0068. The diagnosis of asthma was based on Reddel et al. (12) and Global Initiative for Asthma (GINA) (13), that is, it characterized by recurrent episodic respiratory symptoms such as wheeze, shortness of breath, chest tightness, and cough, besides the presence of reversibility and variability of symptoms together with variable expiratory airflow limitation. Patients were included sequentially during the period between September/2020 and October/ 2021, on basis of the inclusion criteria: adult patients, clinical and spirometric diagnosis of asthma, atopy investigation, and history of COVID-19 (history and laboratory confirmation).

Atopy was confirmed through the analysis of specific IgE for aeroallergens [house dust mites (HDM), cockroaches, cat and dog dander, and molds] through skin prick test (SPT) and/or serum specific IgE ImmunoCAP® (ThermoFisher Scientific, Uppsala, Sweden) (14). The skin prick test used common standard aeroallergens extracts (International Pharmaceutical Immunology–IPI-ASAC, São Paulo, Brazil), including the use of positive (histamine dihydrochloride 10 mg/mL) and negative (glycerin 50.0%) controls (15). Total serum IgE was also assessed (reference values were between 0 and 100 IU/mL).

All patients had, in addition to the characteristic symptoms of asthma, reversibility in at least one spirometry during follow-up at our service. Spirometry was assessed by means of a Koko spirometer (PDS Instrumentation, Louisville, CO, USA), which provides flow-volume and volume-time curves. The techniques and interpretation of the results were those recommended by the 2005 American Thoracic Society consensus and the 2005 European Respiratory Society consensus (16, 17). The reference values used were those established for Brazilians (18). The tests were repeated 15 min after the inhalation of 400 mcg of salbutamol *via* a metered-dose inhaler with a spacer. The bronchodilator response was defined as an increase (over baseline) of at least 12% and 200 mL in forced expiratory volume in one second (FEV1) and/or forced vital capacity (FVC) (16, 17). The 2021 GINA guidelines classifies asthma severity based on the level of treatment required to control symptoms and exacerbations, into steps 1 to 5 (12, 13).

Patients were evaluated for current age, age at onset of asthma, duration of asthma, severity of asthma, presence of atopy and total serum immunoglobulin (IgE). Several comorbidities including diabetes mellitus, hypertension, dislipidemia, obesity, thyroid diseases, gastroesophageal reflux disease (GERD), bronchiectasis, asthma-chronic obstructive pulmonary disease [COPD] overlap (ACO), and oncological pathologies were assessed as well. Only comorbidities with diagnosis confirmed by a specialist or an altered complementary exam were considered.

In addition, asthmatic patients were stratified according to phenotypes T2 or non-T2 inflammation. T2 inflammation was considered for atopic patients, for asthma patient's steps 1 to 4 of GINA with peripheral eosinophils above 300 cells/μL, and for asthma step 5 of GINA, peripheral eosinophil above 150 cells/μL (19).

The history of COVID-19 was assessed during the first face-to-face consultation after pandemic lockdown. COVID-19 was confirmed through a history of suggestive symptoms of the disease, including systemic symptoms, and also a positive diagnostic testing for COVID-19. That investigation included a molecular detection of the viral genome (RNA detection by PCR) and/or serological detection, IgM or IgG against the virus detected around between days 3 and 7 from the onset of symptoms (20).

The clinical manifestations of COVID-19 were also assessed and the asthmatic patients were classified into asymptomatic and symptomatic. Further asthmatic patients with COVID-19 were classified according to the history of asthma exacerbation post-COVID-19. Asthma worsening was based on the maintenance of asthma symptoms after 30 days post-COVID-19 diagnosis associated with a reduction in the Asthma Control Test (ACT) questionnaire, and the need to increase the dose of their maintenance therapy for asthma. ACT score range from 5 (poor control of asthma) to 25 (complete control of asthma), and the minimally important difference (MID), that means the smallest change or difference in an outcome measure that is perceived in the patient's medical management, is three points (21). Chest computed tomography performed during the acute phase of COVID-19 was also evaluated, if the patient had one.

Regarding COVID-19 vaccination, patients received the vaccines according to the government recommendations. CoronaVac vaccine was introduced in February 17^th^, 2021; Oxford, AstraZeneca in March 21^st^, 2021; and BioNTech, Pfizer in April 30^th^, 2021. Table 1 shows the vaccination schedule recommendations (22).

**Table 1:** COVID-19 vaccination schedule for adults (22).

**Month-2021**	**Age group (years)**
January, 17	Health worker, indigenous
February	80 or more
March	69 or more
April	63 or more, and special conditions^**#**^
May, 06	60–62
May, 10 to June, 11	Several comorbidities, and special conditions^*^
June, 16 to June, 29	43–59
June, 30 to July, 14	40–42
July, 15 to July, 29	30–36
July, 30 to August, 4	29–18
August, 5 to August, 16	18–27

# Public security and prison administration professionals and education professionals from 47 years of age.

* Down syndrome (18 to 59 years old); renal patients on dialysis (18 to 59 years old); immunosuppressed transplant recipients (18 to 59 years old); subway and rail (train operators of all ages; other workers in the sector aged 47 and over); pregnant and postpartum women with comorbidities (over 18 years old); people with permanent disabilities (55 to 59 years old); people with comorbidities (55 to 59 years old); pregnant and postpartum women with comorbidities (over 18 years old); bus drivers and conductors; people with comorbidities and with permanent disabilities (45 to 49 years); people with comorbidities and permanent disabilities (40 to 44 years); air transport professionals; port transport professionals; people with comorbidities and with permanent disabilities (30 to 39 years old); people with comorbidities and with permanent disabilities (18 to 29 years); education professionals (45 and 46 years old); pregnant women and postpartum women without comorbidities (over 18 years old); people with Permanent Disabilities (18 to 59 years old).

### Statistical analysis

Chi-squared test was used to compare nominal non-parametric data including female prevalence, atopy, and frequency of comorbidities, frequency of acute symptoms of COVID-19, and frequency of asthma worsening post-COVID-19. The Chi-square test was also used to frequency of COVID-19 vaccination.

The Mann Whitney test was used to compare ordinal categorical data including current age, age at onset of asthma symptoms, duration of disease and total serum IgE.

The level of significance was set at 5% (*p* < 0.05) for all the tests, and 95% confidence intervals for the group comparisons.

## Results

Two hundred and fifty asthmatic patients had returned to a face-to-face consultation during the period between September 25, 2020 and October 8, 2021. Seven patients were excluded because there was no data about atopy, nine patients had performed no spirometry during the last 2 years, and 26, because there was no data about COVID-19. Then, 208 patients were included in this study. Of them, 165 (79.33%) were women, the mean age was 52.75 years [standard deviation (SD) 17.32 years], mean age at onset of symptoms was 18.81 years (SD 16.86 years), and mean duration of disease, 33.96 years (SD 16.52 years). Of all asthmatic patients, 73.08% had severe asthma, that is, they were on steps 4 or 5 of GINA steps of asthma treatment. The majority, 188 (90.38%), was under treatment with inhaled corticosteroid plus long-acting beta2-agonist (ICS plus LABA), ten patients were with ICS alone, ten patients received biologics (omalizumab, mepolizumab or benralizumb) or tiotropium along with ICS plus LABA, and only one patient needed continuous systemic corticosteroid to control disease. The general characteristics of the patients are showed in Table 2.

**Table 2:** General characteristics of asthmatic patients.

**General characteristics**	**Results**
Gender: female (%)	79.33
Current age (years, mean ± SD)	52.75 (17.32)
Age at onset of symptoms (years, mean ± SD)	18.81 (16.86)
Duration of disease (years, mean ± SD)	33.96 (16.52)
Asthma (steps 4 and 5 of GINA) (%)	73.08
Asthma and COVID-19 (*n*, %)	59 (28.37)
Atopy (%)	79.81
Obesity (%)	40.38
Hypertension (%)	37.50
Dyslipidemia (%)	17.31
Diabetes mellitus (%)	16.35
GERD (%)	41.83
Thyroid disease (%)	14.90
Osteoporosis (%)	12.98
Asthma and COPD overlap (%)	12.50
Bronchiectasis (%)	4.81

SD, standard deviation; GINA, Global Initiative for Asthma; GERD, gastroesophageal reflux disease; COPD, chronic obstructive pulmonary disease.

Allergic asthma was diagnosed in 166 patients (79.81%), and of them, house dust mite sensitization being the most prevalent (96.99%), followed by pet dander (36.14%). Allergic asthmatic patients had other concomitant atopic diseases; rhinitis, conjunctivitis, and dermatitis that were observed in 99.40, 66.87, and 15.06%, respectively. The median total serum IgE was 245.60 IU/mL (ranging from 8.2 to 8310 IU/mL).

Thirty-one patients (14.90%) were current or former smokers with a history of exposition to tobacco of 10 or more pack/years. ACO was confirmed in 26 patients (12.50%). Regarding other comorbidities, GERD was the most frequent disease (41.83%), followed by obesity (40.38%), hypertension (37.50%), dyslipidemia (17.31%), and diabetes mellitus (16.35%). The frequency of comorbidities is resumed in Table 2.

Asthmatic patients were classified according to the inflammatory phenotypes: T2 (eosinophilic) or non-T2 (non-eosinophilic). T2 asthmatic patients were younger, had earlier onset of asthma, and longer duration of the disease than non-T2 patients (*p* < 0.001). Total serum IgE was higher for T2 asthmatic patients (*p* < 0.001). Likewise, patients with asthma T2 had more severe asthma (steps 4 and 5 of GINA) than those with non-T2 asthma, *p* < 0.001. There were no other statistical difference between T2 and non-T2 groups for comorbidities, frequency of COVID-19 and peripheral eosinophils (Table 3).

**Table 3:** Asthmatic patients according to inflammatory phenotypes (T2/ non-T2).

**General characteristics**	**T2 phenotype**	**Non-T2 phenotype**	***P***
Gender: female (%)	78.77	82.76	NS
Current age (years, mean ± SD)	51.53 (17.41)	60.24 (14.96)	<0.01^*^
Age at onset of symptoms (years, mean ± SD)	16.50 (14.98)	33.33 (20.75)	<0.0001^*^
Duration of disease (years, mean ± SD)	35.06 (15.85)	27.04 (19.14)	<0.01^*^
Severe asthma (steps 4 and 5 of GINA) (%)	77.65	41.83	<0.001^#^
COVID-19 (%)	27.37	34.48	NS
Obesity (%)	39.66	37.92	NS
Hypertension (%)	34.64	58.62	NS
Dyslipidemia (%)	16.76	20.69	NS
Diabetes mellitus (%)	15.08	24.14	NS
Total serum IgE (UI/mL) (median, range)	280.30 (10.8–8,310)	94.80 (8.2–1,722)	<0.0001^#^
Peripheral eosinophils (cells/μL) (median ± SD)	261.47 (231.26)	192.41 (150.54)	0.099^#^

SD, standard deviation; GINA, Global Initiative for Asthma.

* Mann-Whitney test.

# Chi-square test.

Regarding oncological pathologies, 3 patients were being followed-up by a specialist, all with breast cancer. There was no statistical difference related to COVID-19 or T2 asthma.

**Figure 1:**
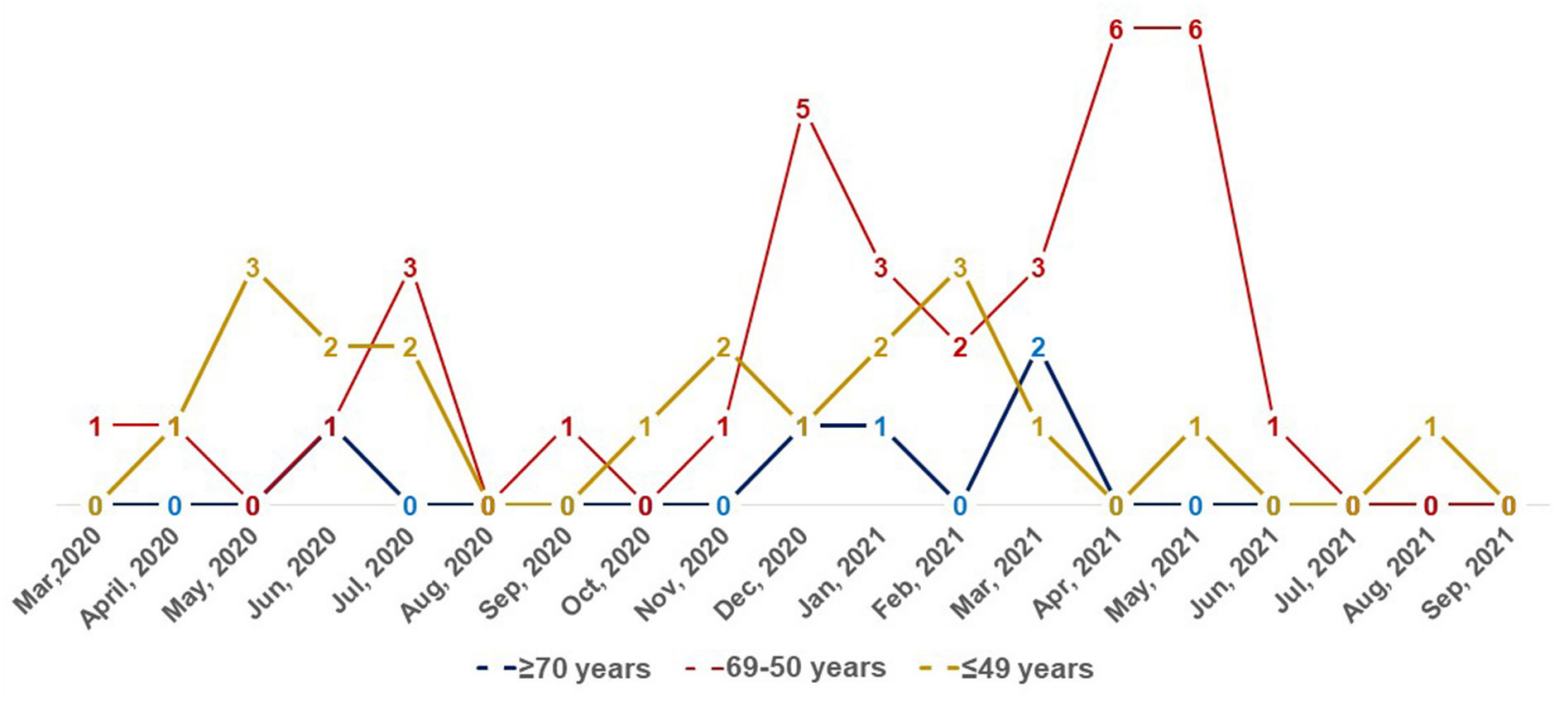
Frequency of COVID-19 (number) in asthmatic patients from March 2020 to September 2021.

Of all patients, 59 (28.37%) had laboratory-confirmed coronavirus disease between March/2020 and September/2021 (Figure 1). Comparing asthmatic patients with COVID-19 and those patients without COVID-19, we observed that there were no differences regarding demographic data, frequency of comorbidities, and severity of asthma. In relation to atopy, asthmatic patients with COVID-19 had more frequently positive specific IgE to cockroach, compared to patients without COVID-19, that is, 20.34 vs. 10.07%, respectively, *p* = 0.047.

Of all asthmatic patients with COVID-19, only seven (11.86%) were asymptomatic, 11 patients (18.64%) had only symptoms other than respiratory, and 41 (69.49%) reported several symptoms in addition to respiratory. The main respiratory symptoms were dyspnea and cough; and systemic symptoms included more frequently fever, headache, adynamia, and myalgia. Of all patients infected with the SARS-CoV-2, eleven (18.64%) needed hospitalization for no more than a week, and required oxygen supply with an O2 mask. None patient required mechanical ventilation.

**Figure 2:**
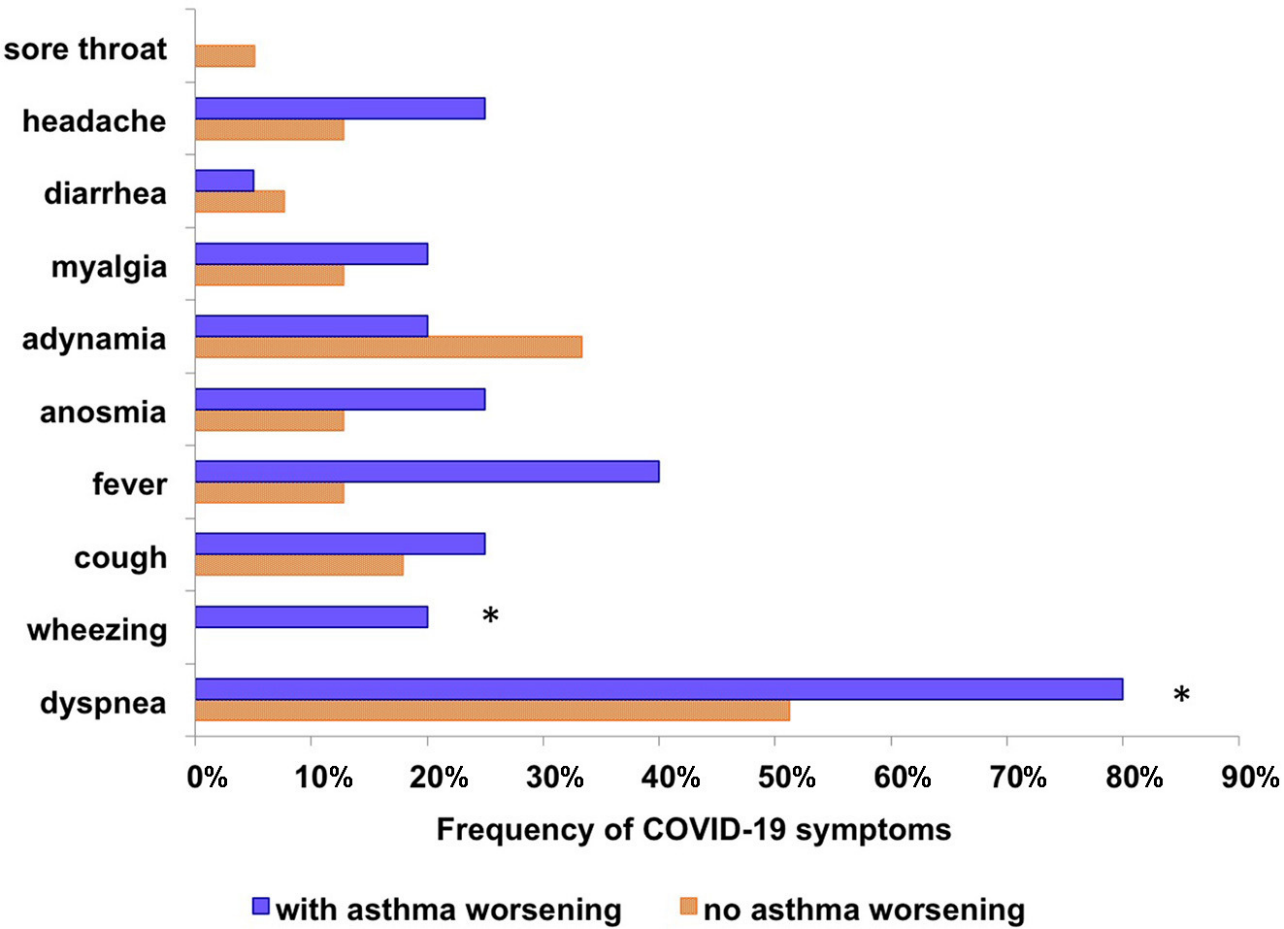
Comparison of the frequency of acute symptoms of COVID-19 between asthmatics with post-COVID-19 respiratory symptoms and asthmatics without post-COVID-19 respiratory symptoms. *p < 0.05, *Chi-square test.

Comparison of asymptomatic and symptomatic COVID-19 in our asthma patients showed no differences related to demographics, comorbidities, or asthma severity. However, we observed that patients with various symptoms related to COVID-19 had more frequently worsening of asthma symptoms (*p* = 0.048). The main symptoms related to COVID-19 were shown in Figure 2. The asthma worsening was characterized by a decrease in ACT (≥3 points) and the need to increment in asthma medication to control disease.

Twenty-three patients with asthma and COVID-19 (38.98%) performed chest-computed tomography (CCT) during acute symptoms. Of them, 13 CCT showed changes related to SARS-CoV2-pneumonia, ranging from 10 to 50% of the lungs, with a similar frequency for both groups of patients, with or without asthma worsening. Other important findings as such emphysema or bronchiectasis were observed in one patient each.

Of 59 asthmatic patients that had COVID-19, 20 patients (33.90%) reported an asthma worsening post-COVID-19. All of them maintained their asthma symptoms, showed a reduction in ACT assessment and had improvement in symptoms after increment of asthma medication. Seventeen patients (11.41%) without COVID-19 reported an asthma exacerbation at some point during the follow-up, from September 2020 to October 2021, much less frequently than asthmatics with COVID-19 (*p* < 0.001).

**Figure 3:**
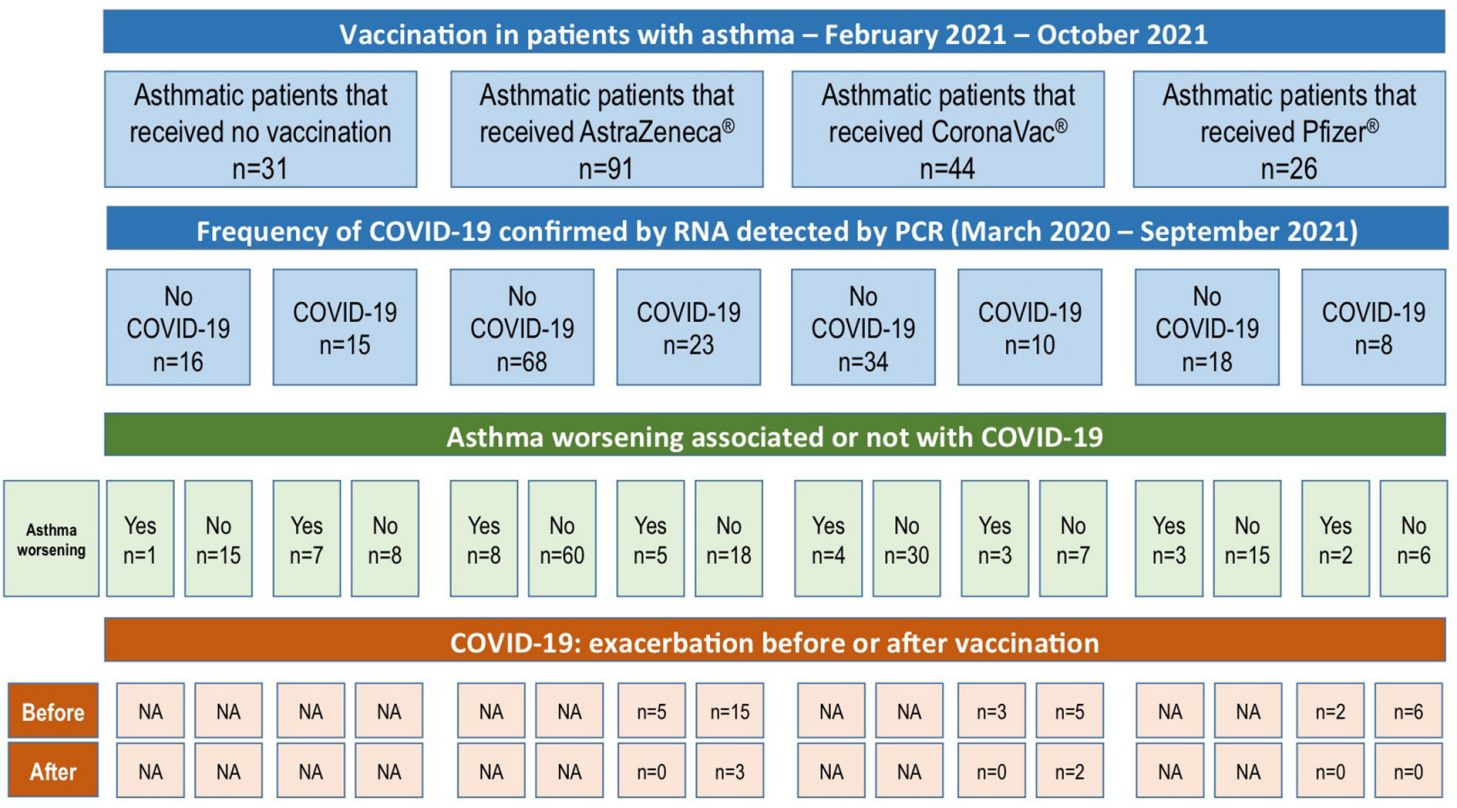
Frequency of COVID-19 and COVID-19 vaccination in asthmatic patients, and frequency of asthma worsening after COVID-19 (before or after vaccination).

The COVID-19 vaccination in São Paulo-Brazil started on January 17^th^, 2021 for health worker and indigenous. The COVID-19 vaccination schedule for adults can be observed in Table 1. At the end of this study, in October/2021, 177 patients had received some COVID-19 vaccine: CoronaVac, 44 patients; AstraZeneca, 91 patients; Pfizer, 26 patients; and 16 patients received some vaccine, but there was no information about them in the electronic medical record. Six patients reported adverse events (headache, fever and/or dyspnea) after COVID-19 vaccination; those symptoms were mild and transitory. Figure 3 shows the frequency of vaccination, the frequency of COVID-19 according to each vaccine group, also the frequency of asthma worsening and if this worsening occurred before or after vaccination [16 asthmatic patients (7.69%) with vaccines without identification in the electronic medical record were excluded].

Patients that received CoronaVac (vaccine) were older (*p* = 0.0004), predominantly female (*p* = 0.028), and had less frequently allergic asthma (atopy) (*p* > 0.05); these characteristics are due to the priority for the application of vaccines in the elderly and, in our country, the CoronaVac was the first vaccine available for the population (Table 4).

**Table 4:** Characteristics of asthmatic patients according to the vaccine (*n* = 161).

**General data**	**CoronaVac**	**AstraZeneca**	**Pfizer**	***P***
Patients (*n*)	44	91	26	NA
Female (%)	90.91	70.33	73.08	0,028^#^
Current age (years, mean ± SD)	61.66 (16.19)	50.26 (17.00)	48.46 (17.47)	<0.001^*^
Age at onset of asthma (years, mean ± SD)	25.50 (26.10)	17.91 (15.68)	16.15 (13.95)	NS
Atopy (%)	72.73	82.42	84.62	NS

SD, standard deviation; GINA, Global Initiative for Asthma.

# Chi-square test.

* Mann-Whitney test.

Of 59 asthmatic patients that had COVID, the asthma worsening post-COVID-19 was reported by 20 patients (33.90), and of them, only five patients had COVID-19 after some vaccination (two patients after CoronaVac and three, after AstraZeneca), although, no asthma exacerbation was reported (Figure 3).

## Discussion

This study assessed asthmatic patients followed up in a tertiary service, from September/2020 to October/2021, as soon as our outpatient clinic returned to face-to-face consultations. Two hundred and eight patients were included in this study; most of them were female with a mean age of 52.75 years and most were atopic and had asthma at steps 4 or 5 of GINA recommendations for asthma treatment. Patients with asthma T2 represented 86.06% of all patients and they were younger, had a longer duration of asthma, and more severe asthma, with a higher frequency of severe asthma on steps 4 and 5 of GINA compared to non-T2 asthmatic patients.

COVID-19 is a severe acute respiratory disease, also associated with other severe systemic manifestations, which can lead to severe respiratory complications and death. Therefore, it would be natural to think that severe asthma would be considered a great risk for infection by the new coronavirus, as well as for complications of this infection. However, the prevalence of asthma in patients with COVID-19 is lower than expected, especially when compared with other comorbidities such as diabetes (23).

Several studies have shown that asthmatic patients under adequate treatment and good control of the disease had a lower risk of severe manifestations of COVID-19 (24, 25). Aveyard et al. (24) showed that patients with asthma who also had a diagnosis of COVID-19 were older, predominantly female and had higher prevalence rates of comorbidities than asthmatic patients without COVID-19. They also found that the number of patients with asthma using ICS was significantly lower in individuals requiring hospital admission.

Rezende et al. (26), assessed data from a nationwide, household-based survey in Brazil [National Health Survey (PNS) in partnership with the Brazilian Institute of Geography and Statistics (IBGE)] and they showed that moderate to severe asthma was not associated to a risk factor for severe COVID-19. They included 51,770 adults who responded to the questionnaire about medical diagnosis and lifestyle risk factors, and they observed that asthma was associated to only 1.5% of severe COVID-19.

In our study, fifty-nine patients (28.37%) reported COVID-19 since the onset of the pandemic. Our asthmatic patients with COVID-19 reported only mild to moderate symptoms related to COVID-19, and only 11 (18.64%) needed hospitalization and required O2 through facial mask. No one needed intensive care unit or orotracheal intubation. There was no difference regarding demographic data, comorbidities, or asthma severity between the groups of asthmatic patients with or without COVID-19. This probably was a consequence of our homogeneous group of patients followed-up in a tertiary center. Most of our asthmatic patients had asthma at steps 4 and 5 of GINA and were under treatment with moderate/high dose of inhaled corticosteroids plus LABA. An interesting data was that the frequency of sensitization to cockroach was higher for asthmatic patients with COVID-19. Some studies suggested an association between COVID-19 incidence and mortality with subindices of socioeconomic status (27, 28). Likewise, some studies showed that sensitization and exposure to cockroach allergens were associated with asthma severity, especially among lower socioeconomic groups (29, 30).

Moreover, patients with poorly controlled asthma are prone to exacerbate asthma and viral infection are an especially important factor, moreover, good adherence to asthma treatment and a correct inhaler technique are essentials for a good control of asthma. During the current COVID-19 pandemic, it was not uncommon for patients to lose their medical follow-up and, consequently, a predisposition to lose control of asthma (31).

In this study, during the period of 1 year, of 208 asthmatic patients, 59 (28.37%) had COVID-19, and, during its acute period, respiratory symptoms were reported by more than 60% of them, although, those symptoms were mild to moderate. Of them, 20 patients (33.90%) progressed to asthma worsening; and needed an increment in asthma medication to control the disease for an average of 6.14 months. It is worth remembering that, in the post-COVID-19 scenario, the maintenance of respiratory symptoms may be a consequence of pulmonary involvement by the virus. However, in our study, patients who reported maintenance of post-COVID-19 asthma symptoms showed clinical improvement, based on clinical history along with ACT assessment, after increasing their previous asthma medication. ACT is an important tool for assess asthma control, Crimi et al. (32) examined and compared patients' and physicians' perceptions of asthma control, simultaneously using the ACT. They encouraged physicians to better explore patients' perspectives in terms of asthma symptoms' perception and control during their complete assessment.

Several comorbidities have been associated with an increased risk of severe COVID-19 or worse post-COVID-19 evolution. These comorbidities include diabetes mellitus, hypertension, COPD, heart disease, obesity, among others (11, 33). In our study, the most frequent comorbidities were GERD, obesity and hypertension, although the frequency of any comorbidity was similar in both groups, patients with COVID-19 or without COVID-19. The presence of asthma-related comorbidities, such as COPD and bronchiectasis, could worsen the respiratory condition of asthma (33, 34). In our study, the frequency of bronchiectasis and COPD was 4.81 and 12.50%, respectively, with no difference between patients with or without COVID-19.

Regarding vaccination, at the time of this manuscript, 177 patients were vaccinated against COVID-19; AstraZeneca vaccine was the more frequent one. Thirty-one had not yet received any vaccine due to the vaccination schedule. Only six patients reported adverse events (headache, fever and/or dyspnea) after COVID-19 vaccination though those symptoms were mild and transitory. The majority of our asthmatic patients had COVID-19 before its vaccination. The only five patients that had COVID-19 after vaccination did not evolve to asthma worsening, although two of them presented some alteration on chest CT (around 15%) previously.

A retrospective study has several advantages as it allows the analysis of a large number of patients and is often more likely to create more uniform inclusion criteria. However, data would be lost due the impossibility of interviewing patients. In our study, it would be important to include more data about vaccination, comorbidities and information on death from COVID-19.

This retrospective study allowed us to assess the evolution of our asthmatic patients who had COVID-19 and compare them with those who did not have COVID-19. After analyzing these data, we observed that although patients with COVID-19 had mild symptoms of the disease, they had worsening asthma symptoms for several months and required modification of the baseline treatment for asthma control.

In conclusion, it should be difficult to assess if asthmatic patients with asthma worsening post-COVID-19 belongs to a group named “post-COVID-19 condition” or they represent a phenotype of asthma that get worsen after COVID-19. According to WHO (7), post-COVID-19 condition occurs usually 3 months from the onset of COVID-19. In this study, we found that COVID-19 was frequent in a group of patients with severe asthma, although, respiratory symptoms were mild to moderate and only few patients needed hospitalization and O2 support. However, a subgroup of these asthmatic patients with COVID-19 had chronic worsening of asthma symptoms and a need for increment asthma treatment and until the end of this study, the need for this dose increase was maintained for an average of 6.14 months.

## Data availability statement

The original contributions presented in the study are included in the article/supplementary material, further inquiries can be directed to the corresponding author/s.

## Ethics statement

The studies involving human participants were reviewed and approved by Comissão de Ética para Análise de Projetos de Pesquisa (CAPPesq) (Approval No. CAAE: 55761122.3.0000.0068). Written informed consent for participation was not required for this study in accordance with the national legislation and the institutional requirements.

## Author contributions

RA: conception and design of the study, acquisition of data, analysis, interpretation of data, and writing the article. NM: acquisition of data. AM and PG-B: revising the article critically for important intellectual content. JK and RA: final approval of the version to be submitted. All authors contributed to the article and approved the submitted version.

## Funding

This work was funded by the INCT/CNPq Project - iii, process number 465434/2014-2.

## Conflict of interest

The authors declare that the research was conducted in the absence of any commercial or financial relationships that could be construed as a potential conflict of interest.

## Publisher's note

All claims expressed in this article are solely those of the authors and do not necessarily represent those of their affiliated organizations, or those of the publisher, the editors and the reviewers. Any product that may be evaluated in this article, or claim that may be made by its manufacturer, is not guaranteed or endorsed by the publisher.
